# Enhancing green bean crop maturity and yield prediction by harnessing the power of statistical analysis, crop records and weather data

**DOI:** 10.1371/journal.pone.0306266

**Published:** 2025-03-10

**Authors:** Miranda Y. Mortlock, David Carey, Hamish Murray, Peter J. Baker, Paul G. Corry

**Affiliations:** 1 Center of Data Science, Queensland University of Technology, Brisbane, Queensland, Australia; 2 Affiliate of QAAFI, University of Queensland, St. Lucia, Queensland, Australia; 3 Food Agility Cooperative Research Centre Ltd., Sydney, New South Wales, Australia; 4 Department of Agriculture and Fisheries, Brisbane, Queensland, Australia; 5 School of Mathematics, Queensland University of Technology, Brisbane, Queensland, Australia; Nepal Agricultural Research Council, NEPAL

## Abstract

Climate change impacts require us to reexamine crop growth and yield under increasing temperatures and continuing yearly climate variability. Agronomic and agro-meteorological variables were concorded for a large number of plantings of green bean (*Phaseolus vulgaris* L.) in three growing seasons over several years from semi-tropical Queensland. Using the Queensland government’s SILO meteorological database matched to sowing dates and crop phenology, we derived planting specific agro-meteorological variables. Linear and nonlinear statistical models were used to predict duration of vegetative and pod filling periods and fresh yield using agro-meteorological variables including thermal time, radiation and days of high temperature stress. High temperatures over 27.5∘C and 30∘C in the pod fill period were associated with a lower fresh bean yield. Differences between specific bean growing sites were examined using our bespoke open source software to derive agro-meteorological variables. Agronomically informed statistical models using production data were useful in predicting time of harvest. These methods can be applied to other commercial crops when crop phenology dates are collected.

## Introduction

Green beans are grown in many parts of the world, and are known as snap beans, wax beans or French beans. They are grown for both the dried bean yield, and increasingly for the nutritious immature pods, which is the interest of this study in Queensland. The green bean (*Phaseolus vulgaris L.*) is a unique crop as it has two separate centres of domestication which are reflected in its genetic diversity and requirement for a warm temperate climate. Two domesticated gene-pools are identified as Andean and Meso-America with further diversity found in wild accessions in other parts of South America [1, 2]. The climate at these locations of domestication informs climatic requirements of the species.

Green beans are grown in Australia’s temperate and semi-tropical regions where production of high quality fresh beans are delivered to consumers and wholesalers. An extended production period is facilitated by using several complimentary geographical locations with a range of sowing dates.

In Australia a short determinate bush green bean (type I) is grown to maturity and pods mature in a window of one to three days [3]. The beans are planted into an irrigated and well fertilized field and mechanically harvested from ten weeks after sowing at optimum pod weight and quality. This project provided a unique opportunity to examine thermal time and other agro-meteorological variables with a large set of field observed crop phenology dates and fresh bean yield. A Queensland company wished to add value to a data asset of past growing seasons. We combined the data with daily meteorological data to derive agro-meteorological variables such as thermal time for pod filling to use in modelling.

In this study, growth and development of a large number of bean sowing dates were of interest and in contrast to many studies on dry beans the focus is on optimum fresh weight of high quality fresh green beans. Many crops are grown for grain or dry beans and the research literature for green pod growth of *Phaseolus* is somewhat limited. Our novel insights will be particularly useful for producers. Australia produced *$*78 million of fresh beans in 2016–17, up from *$*69 million in 2015–16. Queensland is the largest producer and Tasmania and Victoria also produce green beans [4].

Boote and Scholberg [5] noted it was not possible to accurately use simulation modelling for predicting pod fresh weight because the product is composed of 90*%* water. Model outputs of dry biomass do not account for the fact that pods are 90 to 95*%* water. Pod fresh weight is a function of photosynthate supply to the immature pods and changes in pod composition over the period. Fresh weight and stringiness increases with pod maturity which affect pod market quality and dictates the time of harvest to optimise both weight and tenderness. Stolle-Smits, Beekhuizen et al. [6] found at 14–23 days after flowering the pod reached maximum pod length and a maximum of 91*%* water content. The Australian Agricultural Production Systems sIMulator (APSIM) is a comprehensive model developed to simulate biophysical processes in agricultural systems, and the green bean module could not be used for modelling marketable fresh weight and pod quality [7, 8]. Process based crop models, such as APSIM, predict dry matter using time, efficiency of water use and photosynthetically active radiation (PAR), are sophisticated and increasingly valued for research. In simulation models dry matter accumulation is calculated using radiation use efficiency, this is affected by temperature and water stress [9].

**Fig 1:**
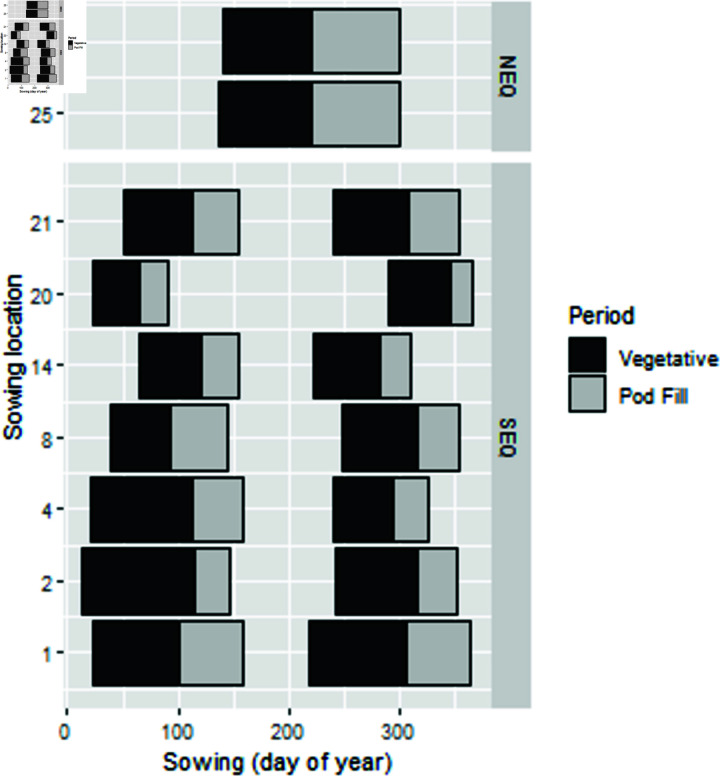
Green bean growing periods for NE and SE Queensland.

Using Australian open source weather data and our custom built R package [10] we derived a range of agro-meteorological variables to describe relationships of agronomic and agro-meteorological data in predicting duration of growth and fresh bean yield. With this unique dataset we developed agronomically informed parsimonious models with useful predicative ability. Statistical modelling has used data from meteorology for prediction [11]. This approach is an empirical linear or non linear modelling approach as compared to a process based model such as in APSIM [12]. The bean growing seasons are shown in Fig 1.

### Climatic conditions for bean production

Green beans have a short growing season so a range of sowing dates result in a long production season with multiple harvests. In South East Queensland (SEQ) there are two sowing seasons: from October to December (‘Spring’), from January to May (‘Autumn’) and in Bowen, North East Queensland (NEQ) a third, ‘Middle’ season occurs from May to October (Table 1).

**Table 1:** Field conditions of bean production in Queensland.

Location	SE Queensland	SE Queensland	North Queensland
Season	Autumn	Spring	Middle
Observations	549	585	367
Soils	black soils	black soils	deep well drained
Sowing Months	Mar Apr May	Aug Sep Nov	May to Oct
Population (plants /ha)	250,000	250,000	250,000
Irrigation, various	optimum	optimum	optimum
Harvest	Mechanical	Mechanical	Mechanical

**Fig 2:**
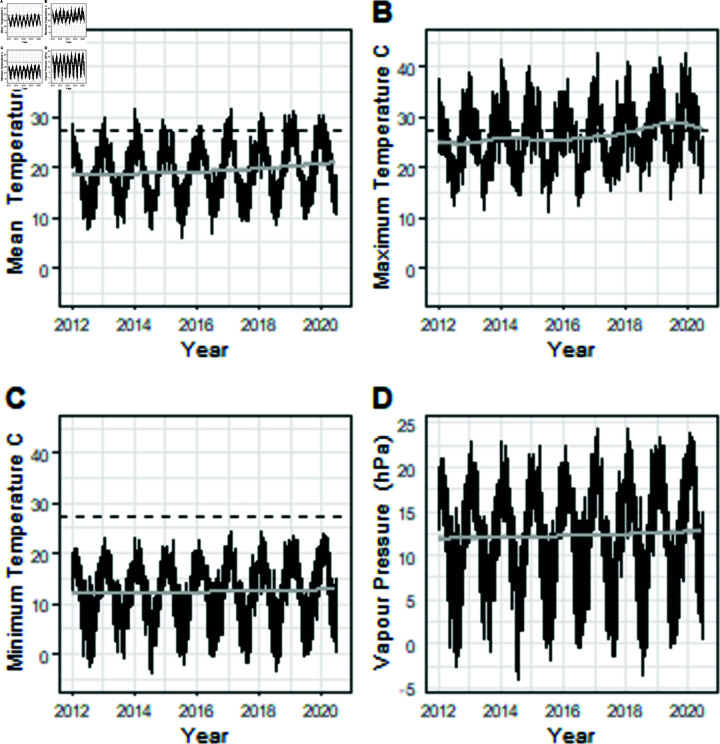
Daily temperatures with reference line at 27.5 ∘C and vapour pressure for a SE Queensland location with trend line. (A) Daily Mean Temperature (∘C). (B) Daily Maximum Temperature (∘C). (C) Daily Maximum Temperature (∘C). (D) Vapour Pressure (hPa).

Examination of the temperatures and vapour pressure data at growing locations across years show an increase in maximum temperature over the last ten years (Fig 2).

**Fig 3:**
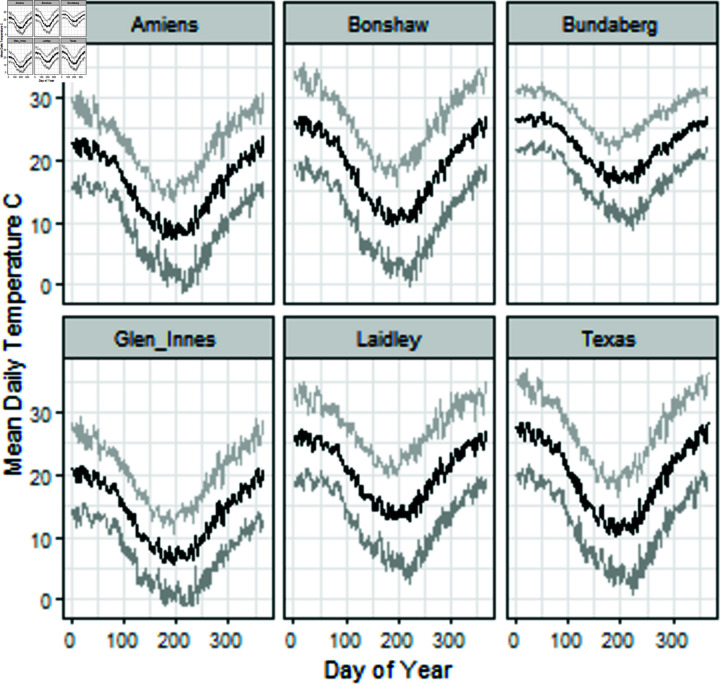
Mean, min and max daily temperatures for selected locations (medians from 2012 to 2021).

Locations in Queensland and northern New South Wales had varied temperature profiles (Fig 3). For each historic sowing the daily weather was extracted and matched to the sowing date and location. Using the crop dates of sowing and petal fall agro-meteorological variables were derived and used in the modelling.

Under well watered conditions intercepted radiation and temperatures are important and so we used these variables in our models. Early emergence and optimum leaf cover in the vegetative period intercepts sunlight for leaf growth, flowering and pod filling. Type I green beans are considered photo period insensitive and there is a simple relationship with thermal time and development [3]. The principles of crop light interception show primary productivity of plant biomass *P_n_* is succinctly described by the following [13, 14]:


$$\begin{array}{ccc} P_{n}=S_{t}\epsilon_{i}\epsilon_{c}∕k & & \end{array}$$ (1)

Primary production *P_n_* is above ground biomass(dry weight per area) over the growing period for a particular site. It is a function of the annual incident solar radiation *S_t_* which is determined by site and year as solar radiation (*MJm*^−2^) and varies with latitude and time of year. The efficiency of radiation interception by the crop is denoted by *ϵ_i_*, the efficiency of conversion into biomass *ϵ_c_* and *k* is the energy content of the plant mass in units of *MJg*^−1^. The radiation use efficiency of faba bean was found to be 1.03 to 1.29 *gMJ*^−1^ [15].

Yield potential of a crop is a function of interception of light and the transfer of the products of photosynthesis into plant biomass and the component of interest (e.g pods). Yield potential *Y_p_* is defined by Evans [16] as ‘the yield of a cultivar when grown in environments to which it is adapted, with nutrients and water non-limiting, and with pests, diseases, weeds, lodging and other stresses effectively controlled.’


$$\begin{array}{ccc} Y_{p}=\eta .P_{n} & & \end{array}$$ (2)

where the harvest index *η*, (*η* ≤ 1) is the efficiency with which biomass is partitioned into the harvested product. From these principles, the main factors which influence yield are:

- early canopy development and an optimum green leaf area,
- biotic stress pests and diseases can reduce the green leaf area and pod yield,
- abiotic stresses (temperature stress, water stress, saturated soils or nutrient stress) can reduce yield and quality, and
- harvest time for optimum weight and quality of fresh bean pods (e.g. pod stringiness reduces quality).

Indices of growth assess the environmental potential for productivity when there is no water limitation [17]. A growth factor to account for the extent of radiation interception RIX (RIX  ≤ 1) was derived from research data on biomass production. Then a growth index *I_g_* which quantifies potential growth per unit of crop development, was calculated for a site using weekly values of daily incident radiation *R_i_* and mean daily temperature *T_i_* are the mean daily solar radiation and the mean daily temperature for week *i*.


$$\begin{array}{ccc} I_{g}=\frac{R_{i}}{T_{i}-T_{b}}.RIX & & \end{array}$$ (3)

This equation is used for weekly data across the growing period of the crop, and requires Leaf Area Index (LAI) measurements. LAI is a unitless measure of the ratio of the area of green leaf area for a unit land area, and it is required to be over approximately 2 to 3 for full radiation interception (RIX = 1).

In crop studies green leaf area, light interception and biomass (dry weights) of above ground plant parts are measured on several occasions and at key phenological stages throughout the season. Such studies are resource intensive and form part of a research program as several sites and years are required to provide data. As an efficient alternative, our contribution uses statistical modelling with a large data set of bean phenology, derived agro-meteorological variables and fresh yield.

### Temperature and growth

Monteith [14] introduced the concept of thermal time *ρ* which expresses time on the developmental clock of a plant as the summation of temperature above a threshold. The laying down of the leaf and flower nodes, the plastochron development, is a function of the thermal time and temperatures experienced within the early growth stage. Biomass yield is a function of photo-synthetically active radiation (PAR) interception and yield is improved by early green leaf cover, where water is not limiting.

From the literature, the growth curve for bean dry weight appears as a characteristic logistic curve, which is typical of many plant growth curves under well-watered conditions. Cardinal temperatures (base, optimal and maximum) of the developmental process were found to be 6.8, 24.0 and 34.1∘C, when hourly temperatures, in the phase between emergence and the appearance of the first bean flower, were used [18]. Temperature sensitivity occurs at phenological stages such as petal fall and during pod fill. Partitioning of dry matter studies on beans used a base temperature of 5∘C [3].

Further studies showed bean growth stages had a base temperature of 4.2∘C, an optimum of 24∘C and ceiling of 30∘C, and mild water deficits were not assumed to have any affect on development [19]. The vegetative period ends at petal fall and actual start date of the pod growth will vary with the determinacy of the variety. Across four bean cultivars the mean time required to develop a node was 6.1 days at 18∘C, 3.0 days at 23.3∘C, and 0.91 days at 28.3∘C. Supra-optimal temperatures for a crop either as high day or night temperature can reduce yield. A higher temperature tended to decrease time to flowering by enhancing the rate of vegetative development, while it tended to increase the days to flower by enhancing the photoperiod gene activity. This resulted in a U-shaped curve of time to flowering in response to temperature.[20].

High night temperature is detrimental to growth of many crop species and in particular affects many traits in the reproductive phase [21]. High night-time temperatures appeared to have larger effects than maximum daytime temperatures. High temperatures during pod fill may reduce reproductive performance when tolerance thresholds are exceeded. Bean flowers and developing pods drop in high temperatures thus reducing yield.

Green bean as a C3 warm temperate crop, will lose more biomass in the process of photo-respiration when temperature increases compared to a C4 tropical crop [13]. As climate changes and temperatures increase producers will be looking to better manage these stresses in bean production. The anomaly for Eastern Australia and Queensland temperatures are in the order of a 2∘C for both the minimum and maximum temperature in each of the bean growing seasons [22].

When calculating thermal time, we used a base temperature of 5∘C and a maximum temperature of 30∘C for field grown beans using daily temperatures [7, 23].

## Objectives

This study utilised unique and detailed crop phenology records of commercial bean sowing and flowering dates from sites in the Lockyer Valley and North Queensland (Table 1). Specific agro-meteorological variables were derived for over a thousand commercial bean planting dates. With this data we aimed to:

- use statistical summaries and predictive modelling to gain insights from specific agro-meteorological variables to estimate growth duration of time to petal fall, duration to pod harvest date and fresh bean yield,
- examine median values of agro-meteorological variables such as thermal time and stress temperatures experienced by sowing date and location using a ten-year data set, and
- sample fresh weights in the field to estimate and fine tune yield estimations.

## Methods

Dates of sowing, petal fall and harvest for bean crops were recorded by a Locker Valley speciality green bean producer. Dates of sowing and petal fall were recorded for producing fresh marketable green beans.

Daily climate records are available from Queensland Government’s Department of Environment and Science. This database consists of daily climate data for Australia from 1889 to present, in a number of ready-to-use formats, suitable for modelling and research applications [24]. These SILO products are provided free of charge to the public for use under the Creative Commons Attribution 4.0 license and a fair-use limit is imposed. An R package *bomrang* uses data from the Australian Bureau of Meteorology [25] and another R package calculates weather over critical time windows for ecological studies [26]. As part of this project, we developed an R package *cropgrowdays* to derive agro-meteorological variables when crop phenology and daily weather data are available.

### Bespoke *cropgrowdays* R Package

The R package *cropgrowdays* was developed to compute agronomically informative variables using SILO meteorological data [10]. These included agro-meteorological data items such as thermal time (also known as growing degree days) within a period, and mean daily or cumulative radiation within a given period. Currently, functions are provided for calculating growing degree days, stress days, cumulative and daily means of radiation data, and assisting in various day of year units. The package is available under Creative Commons 4.0 licence (see S1 File). Historical meteorological data were extracted from SILO [27] and matched to each sowing and the following agro-meteorological variables were calculated:

- Thermal time as growing degree days (∘C d),
- Minimum, average and maximum temperatures (∘C),
- Cumulative radiation (*MJm*^−2^),
- Average daily radiation (*MJm*^−2^),
- Stress days (number of days above two temperature thresholds), and
- Vapour pressure (*hPa*).

### Agronomic and agro-meteorological variables

The agronomic data consisted of date of sowing, date of petal fall, date of harvest, location and marketable yield. Data were validated, cleaned and vegetative and pod filling growing periods were used to make a range of agro-meteorological variables using *cropgrowdays*. They were constructed for each observed sowing. The historical dataset of sowing dates and crop phenology was assembled to include a range of derived agro-meteorological variables (S1 Table Derived agro-meteorological variables). Therefore, two datasets were constructed using the *cropgrowdays* functions to obtain agro-meteorological variables:

- Agro-meteorological variables for past sowing dates. Vegetative period, pod fill period and fresh yield were modelled using our agro-meteorological predictors.
- A dataset of agro-meteorological variables for all potential past sowing dates for ten years and around 20 planting locations used to make median, 20th and 80th percentile of agro-meteorological variables.

### Statistical methods

We aimed to predict growth periods and fresh green bean yield from combinations of agronomically informed variables developed using sound and practical statistical models. Predominantly, we used growth durations as the focus on predicting durations from localised agro-meterological variables.

**Table 2:** Variables in models for predicting duration of growth periods.

Response	Predictor variables used in models
Vegetative duration (d*)	sowing day (as day of financial year)
(both seasons)	thermal time in vegetative period (∘C d)
	average daily radiation across the period (∘C)
Vegetative duration (d)	sowing day (as day of financial year)
(Autumn season)	thermal time in vegetative period (∘C d)
	average minimum temperature (∘C)
	average daily radiation
	cumulative radiation in vegetative period
	average maximum temperature first 28 d post sowing (∘C)
	average daily radiation first 7 d post sowing
	average daily radiation first 28 d post sowing
	average minimum temperature first 28 d (∘C)
	average vapour pressure first 28 d
	average maximum temperature first 14 d post sowing (∘C)
	average minimum temperature first 14 d post sowing (∘C)
Pod fill duration (d)	thermal time in pod fill period (∘C d)
(both seasons)	days over 30∘C in 5 d post petal fall
	days over 30∘C in pod fill period
	average minimum temperature total growing season (∘C)
	days over 30∘C in 15 d post petal fall
	days over 27.5∘C in 15 d post petal fall
Total duration (d)	sowing day (as day of financial year)
(Autumn season)	cumulative radiation in vegetative period
	average daily radiation in vegetative period
	days over 27.5∘C in vegetative period
Total duration (d)	cumulative radiation in vegetative period
(Spring season)	average daily radiation in vegetative period
	thermal time in vegetative period (∘C d)
	average maximum temperature in vegetative period (∘C)
	average minimum temperature in vegetative period (∘C)

* Abbreviations d = day, thermal time = growing degree days (base 5∘C capped at 30∘C), se = standard error).

Since the 1980s, agro-meteorological variables have been used in predictive plant and crop modelling [17]. The statistical approach employed here balanced the requirement for rigourous and robust statistical modelling while also incorporating the need for practical and readily available agronomic and agro-meteorological variables. (Table 2 and S2 File) outline details of these variables.

Growers are interested in predicting the growing duration given the planting time. Since keeping models as simple and as realistic as possible was one aim of these studies, agronomic variables for inclusion in the initial model were chosen based on informed knowledge of crop growth. For example, sowing day is an important variable for modelling because it is related to whether the plant experienced a declining temperature or an increasing temperature. Since there is little overlap in the two green bean growing seasons in Australia, sowing day also indicates the season. Note also that our data are for the southern hemisphere. We also employed other agronomically useful variables like temperature, degree days and stress days over various periods of growth.

Based on the context, many models may be similar and equally useful. We were conscious of merely using statistical significance and so employed robust statistical procedures and model checking. We used robust statistical modelling, and model simplification based on the principle of parsimony which is when a simpler model with fewer variables is favoured over more complex models with more variables. The iterative statistical process is not trivial, and starts with data validation, model fitting and finally model selection. After data validation, matrix correlation plots were employed to check on linear relationships (correlation). Collinearity was handled by excluding highly correlated variables. Models with a lower Akaike Information Criteria (AIC) indicate a better model fit but, using parsimony, then models with fewer variables are favoured. A final model was determined by stepwise AIC by using groups of variables within relevant growth periods. The final models were obtained using concepts which should prove useful in providing an application for agronomists to replace their complex spreadsheets.

The selected models were based on large datasets and robust statistical methods and, despite the uncertainty of some of the measurements, we were able to fit models useful for prediction. We also provided 80% prediction intervals to assist in capturing the uncertainty for predicting responses.

All data manipulation, wrangling and modelling was completed with R software [28]. The ‘stepAIC’ function from the ‘MASS’ package [29] was employed for robust statistical modelling, and the simplification was based on the principle of parsimony. In essence, as described above, a large number of well chosen agrometerological variables were included in the initial model and the step-wise procedure removed variables which were least related to the outcome variable. This process used a standard, but a well defined, stopping criterion to establish a final model.

Logistic functions are commonly used for describing plant growth. We compared the logistic growth curves of pod fresh weight [6] against thermal time for the seasons. We used the nonlinear least squares function from the nlme R package [30]. The self-starting function ‘SSlogis’ from the ‘nlme’ R package was applied. The parameters are the upper asymptote ‘Asymp,’ a numeric parameter representing the x value at the inflection point of the curve ‘xmid,’ and a numeric scale parameter on the input axis ‘scal.’ In these functions, ‘b’ is replaced by ‘scal’ = -1/b.

### Components of fresh yield

Randomly collected samples from a predetermined location at 9 and 14 days prior to actual harvest, were used to determine commercial yield potential and get statistical estimate of pod components. Commercial packets (200g) of locally grown fresh beans had a mean pod bean weight of 4.91 g. We used statistical estimation of components of yield on a fresh weight basis prior to harvest. Components making up fresh pod yield are:

**Fresh weight yield = Plants per area x Pods per plant x Pod weight**

These statistical estimates required at least 12 to 15 samples to estimate the mean within 10% when simple randomised sampling is used. Due to COVID19 restrictions on travel and field work in Queensland it was not possible to continue sampling in 2021.

**Table 3:** Components of bean pod yield before harvest.

Date of sampling	53 day after sow	45 day after sow
Sample number	11	12
Sampled area (0.5625 *m*^2^	0.75 m row	0.75 m row
Planting Date	19/2/2021	27/2/2021
Petal fall Date	1/4/2021	8/4/2021
Actual harvest Date	22/4/2021	28/4/2021
Duration vegetative	41 days	40 days
Duration pod fill	21 days	20 days
Days after sowing	53 days	45 days
Days after petal fall	12 days	5 days
Days before harvest	9 days	14 days
Plant population (actual) on sample date	172,931	234,077
Pods per plant (mean, se)	15.58 (0.83)	5.28 (0.52)
Pod weight (g) (mean, se)	3.54 (0.08)	0.92 (0.05)
Fresh pod on sample day kg/ha (mean, se)	9268 (318)	1166 (157)
Proportion of pod weight*	0.723	0.187
Proportion of final yield	0.758	0.110
Actual marketable yield (kg/ha)	12,092	10,578
* assuming pod weight 4.9g		

## Results

### The production environment

### Prediction using agro-meteorological inputs

Derived agro-meteorological variables considered in models showed cumulative radiation, days of high temperature and thermal time (calculated as growing degree days with base temperature 5∘C and capped at 30∘C) could assist in predicting duration of growth periods and yield.

A selection of agro-meteorological variables found to have value in modelling are named in Table 2. Predictive models and statistical summaries of agro-meteorological variables were found to be useful in modelling (S2 File).

Many models were built and, in general, the derived variables were useful in describing growth and to a lesser extent fresh yield.

### Field variation of fresh sampled beans

Plants and bean pods were counted and weighed from randomly selected samples on a 0.75 m row length. The proportion of pod weight at sampling date was based on a mature marketable pod of 4.9 g. The plant stand from 45 days to 53 days after planting, showed a decline if it was assumed a similar population was planted. This could also be due to a difference in plant population at seeding or plant establishment, although both planting blocks were at the same site. If this is a true decline in plant number it suggests plant competition has occurred within a row and the resulting plant population is adequate for maximum yield. Over seeding at planting, can occur as a strategy to avoid a low or patchy plant population at harvest, and this may lead to a slight decline in plant population from a good emergence. There were an average of 15.6 pods per plant (se 0.83) at 9 days before harvest.

Table 3 shows the components of yield of the sampled fields. At 14 days pre harvest the pod weight was 11% of final fresh yield and at 9 days pre harvest the pod weight was 75.8% of final fresh yield. The pod size was 18.7% and 72.3% respectively of final yield.

The statistical models applied to the data for these seedings were able to accurately predict the vegetative and pod durations within a day of the observed sowing periods for ground-truthed samples. Agronomist visit fields to ascertain when to harvest and modelling total growing duration may assists in focusing when to make field observations.

Models were applied to fields and observed and predicted durations are shown in Table 4 and described in (S2 File).

**Table 4:** Observed and predicted growing periods (days) of vegetative, pod fill and total duration using linear models. (obs = observed and pred = predicted).

Period	model	19 Feb	27 Feb	19 Feb	27 Feb	difference
		obs	obs	pred	pred	
Veg		41	40			
	modv1	.	.	40.5	39.6	0.5 and 0.4
	modv2	.	.	41.6	40.8	0.6 and 0.8
Pod		21	20			
	modp1	.	.	21.6	20.6	0.6 and 0.6
	modp2	.	.	21.4	20.6	0.4 and 0.6
	modp3	.	.	21.2	19.5	0.2 and 0.5
	modp4	.	.	22.2	19.4	1.2 and 0.6
	modp5	.	.	22.3	21.7	1.3 and 1.7
Total		62	60			
	modtot1		.	63.5	64.3	1.5 and 3.5

### Radiation interception and productivity

Interception of PAR radiation by adequate green leaf cover drives biomass production. Early leaf cover assists in accumulation of total biomass when water is not limiting. We have no measure of LAI but we can use some estimates from the literature. At petal fall we can assume full (100%) leaf cover [31, 32]. A LAI of around 2.5 at 50 days after sowing was measured in a bean crop in Florida [33].

Based on Eq 3 we made a simple productivity index for the vegetative (*I_veg_*) and pod fill period (*I_pod_*) respectively. RIX is set to 1 as we are assuming adequate leaf cover.

Where *R_i_* is average daily radiation and *T_i_* − *T_b_* is average temperature over the vegetative period.


$$\begin{array}{ccc} I_{veg}=\frac{R_{i}}{T_{i}-T_{b}}.RIX & & \end{array}$$ (4)

Where *R_i_* is average daily radiation *T_i_ − T_b_* average temperature over the pod filling period.


$$\begin{array}{ccc} I_{pod}=\frac{R_{i}}{T_{i}-T_{b}}.RIX & & \end{array}$$ (5)

**Fig 4:**
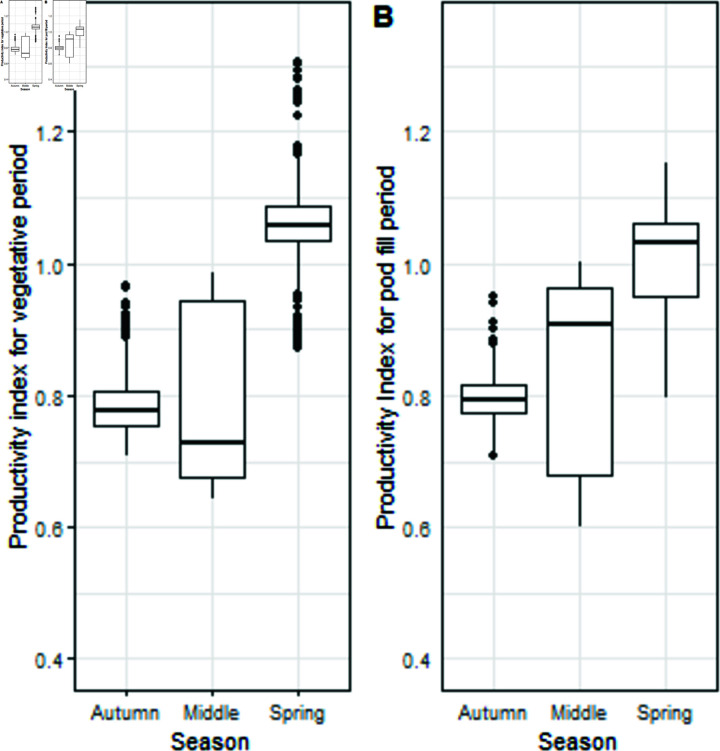
Productivity index for vegetative and pod filling periods by season. (A) Productivity Index for vegetative period. (B) Productivity Index for pod fill period.

Indices were derived to act as a proxy of potential productivity for the two periods of growth (vegetative and pod fill) and the three seasons. The seasons differed with the Spring season potentially more productive than the Autumn season (Fig 4) due to a more favourable radiation to temperature balance. The productivity indices do not incorporate temperature stress.

**Fig 5:**
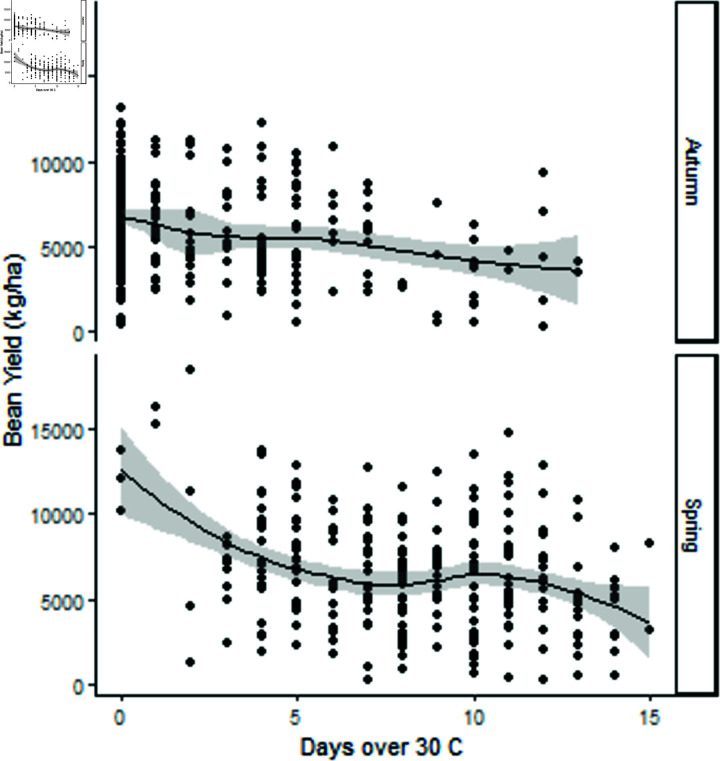
In the first 15 days of pod fill high temperature over 30∘C decreased marketable yield for both seasons.

### Fresh bean yield

Fresh yield prediction, due to the somewhat imprecise measure of marketable fresh weight, was more difficult to model (S2 File). The R squared for models were low. Fresh weight may vary in water content but the measurement after harvest at the packing shed aimed to minimise such differences. We used actual harvest date as our response variable and bean quality was assumed to be marketable by the producer as poor quality crops were not harvested. Quality is of critical importance for maximising the value of harvested green beans. Commercial harvests are planned to occur within a six day optimum harvest window to maximise quality.

It is documented in the literature that once daily maximum temperature exceeds 30∘C, both plant growth rate and yield declines. High temperatures were observed in the pod fill period and these were also associated with a decline in yield as the number of days over 27.5∘C in the pod fill period increase. Hence, a potential high yielding environment will not reach its yield potential where high temperatures occur. We found that days of temperatures over 27.5∘C or 30∘C occurred frequently in Queensland.

Yield declined as the days of high temperature in the pod fill period increased (Fig 5). The productivity indices are an assessment of potential productivity, however it does not take into account the stress days of temperatures over 27.5∘C or 30∘C.

As the crop has a short season, and is harvested at peak quality, this occurs at about 1050, 1080 and 1010 degree days for Autumn, Middle and Spring seasons, respectively (Table 5).

**Table 5:** Growing degree days (GDD) for growth periods and seasons.

Season	Period	number	GDD median	Upper 80th percentile
*Autumn*	Veg	(n = 549)	740	761
*Middle*	Veg	(n = 367)	743	772
*Spring*	Veg	(n = 585)	709	724
*Overall*	Veg	(n = 1501)	731	746
*Autumn*	Pod	(n = 549)	316	333
*Middle*	Pod	(n = 367)	344	364
*Spring*	Pod	(n = 585)	317	326
*Overall*	Pod	(n = 1501)	325	338
*Autumn*	Total	(n = 549)	1050	1070
*Middle*	Total	(n = 367)	1080	1110
*Spring*	Total	(n = 585)	1010	1030
*Overall*	Total	(n = 1501)	1040	1060

Using a three parameter logistic functions, to fit the increase in pod fresh weight from petal fall to harvest, the two seasons had a significantly different growth curve. We fitted thermal times using less than 1080 ∘C d, as above this thermal time pods become stringy and thus fresh weight is past its optimum.

**Table 6:** Coefficients of the logistic model of fresh pod harvest to thermal time after petal fall by season (*Autumn and **Spring).

Parameter	Estimate	Std. Err	t value	p value
Asymp1*	6064	157.4	39	<0.001
Asymp2**	6371	162.0	39	<0.001
scal	22	3.9	6	<0.001
xmid1*	876	6.7	131	<0.001
xmid2**	836	5.9	143	<0.001


**Fig 6:**
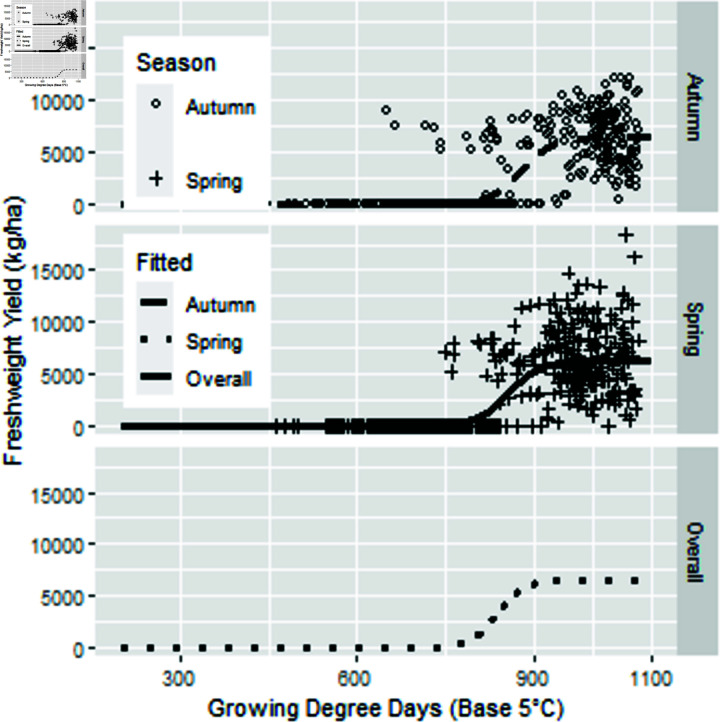
Three parameter logistic model fresh pod yield as a function of thermal time.

The fitted parameters varied significantly by season (p<0.001). The coefficients are shown in Table 6. The best model has separate asymptote, and separate ‘xmid’ but a common ‘scal.’ In Fig 6, there is a large variation in the fresh weight. Quality is not assessed in the field and can reduce the yield by up to 20%. Conditions in transport and at the delivery shed may influence water content and thus weight of fresh beans.

### Agro-meteorological medians

**Fig 7:**
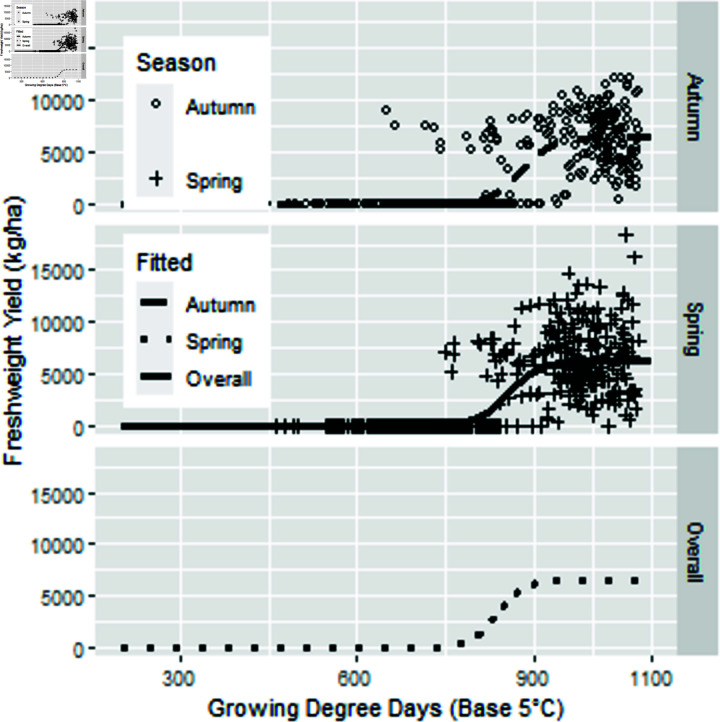
Maximum temperature during pod fill by season.

Agro-meteorological variables plotted for observed historic bean pod filling periods allow insight on temperature stress that may be experienced. The median of a ten year period of maximum temperature of the sites during the pod filling period was plotted. For the sites of bean production in SE Queensland the maximum temperature of over 27.5∘C frequently occurs in each season (Fig 7).

## Discussion

Currently, to develop agronomic models for prediction, we usually conduct multi-year research studies which are expensive due to being resource and labour intensive. Our novel approach employs observed production data and publicly available historic weather data to derive agro-meteorological variables for modelling fresh green beans. Yield is typically measured as dry weight in crop research. However, green bean producers are paid for fresh weight. Models developed here concentrate on traits important for production, namely growing phase durations and fresh weight.

Employing data from over 1500 sowings in Queensland, we estimated growing periods and green bean production using thermal time, radiation and temperature. Using a large production dataset matched to publicly available daily weather, we produced tailor made agro-meteorological variables for each sowing. Given the statistical nature of the modelling, we can quantify the uncertainty in predictions. For instance, predictions of green bean growing duration were estimated within a day of actual maturity. These results could reduce time spent by agronomists in field assessment of bean readiness for harvest.

Models developed also provided other insights around factors affecting fresh bean yield. This highlighted that high temperatures in the pod fill phase reduced yield. In the near future, with an expected 0.5∘C of warming, we can use past data to highlight which locations and sowing dates will likely be affected by higher temperatures.

Dry weight yields were not available, nor relevant, so we modelled green bean fresh weight. Fresh weight yields are harder to model than dry weight yields. The logistic curve of fresh green bean yield in relation to thermal time differed significantly between the Autumn and Spring seasons (p <0.001). While statistically different, the parameters are similar and so may not be different economically. Since the data collection was relatively coarse, there is an opportunity to refine the models by further sampling the production environment. Future studies may expand the approach developed here and use freely available weather data to make “on-farm application for predictions” so that producers may save time and money.

Historic weather data for Australia is available from the Queensland government SILO site. For this project, we developed open source software to both extract the weather data and derive agro-meteorological variables [10]. Employing recent weather data proved useful for modelling. Historic data highlights both the increase in temperatures over the last 100 years as well as the increasing number of days of high temperatures of days above 27.5 ∘C.

## Conclusion

Average global temperatures have increased by about 0.8 ∘C since 1880. Most of that increase appeared in recent times, with two-thirds of the warming occurring since 1975, at a rate of roughly 0.15–0.20 ∘C per decade [34]. Growing a warm temperate green bean crop in subtropical Queensland involves planting in a short season and avoiding high temperatures during bean development.

Publicly available daily weather data and open source software were employed for modelling. A number of agro-meteorological variables in Queensland were derived using an agronomic framework. We added value by applying statistical methods to obtain tailored models for prediction of green bean production.

We demonstrated that statistical models using observed past and current weather were useful for modelling. For planning purposes, these models could be employed for short term prediction by using localised weather forecasts or for long term prediction by using hypothesised weather likely to occur under various climate change scenarios.

## Supporting information

S1 File The cropgrowdays R package. (PDF)

S2 File Statistical models details. (PDF)

S1 Table Agronomic and agro-meteorological variables. (PDF)
