# Supplementary material for: Enhancing green bean crop maturity and yield prediction by harnessing the power of statistical analysis, crop records and weather data
Source: PLoS One. 2025 Mar 10;20(3):e0306266. doi: 10.1371/journal.pone.0306266 (PMC11893118; doi:10.1371/journal.pone.0306266)
Supplement: S1 File — (PDF) [file pone.0306266.s001.pdf]

# S1 The cropgrowdays R package

Version: 0.2.0

## Overview

When modelling crop data, the **cropgrowdays** package provides `R` functions to efficiently calculate agrometeorological quantities of interest. Functions are provided for calculating growing degree days, stress days, cumulative and daily means of weather data. Historic and recent daily meteorological data for all of Australia can be obtained from the Queensland Government's Department of Environment and Science (DES) website https://www.longpaddock.qld.gov.au/. This data is freely available under the Creative Commons 4.0 licence.

In addition, functions are provided to convert days of the year to dates, and *vice-versa*. Day of the year starts at 1 and are numbered from 1 January although any date may be specified if, for instance, the growing season is in the middle of the calendar year.

We recommend using the **cropgrowdays** package in conjunction with the **tidyverse**[1] and **lubridate**[2] packages.

## Installation

In `R`, you can install the latest stable version of **cropgrowdays** via CRAN with:

```
install.packages("cropgrowdays")
```

After installation, to obtain help and details of vignettes, simply use:

```
library(help='cropgrowdays')
```

The development version is available at https://gitlab.com/petebaker/cropgrowdays.

## Details

There are four key agrometeorological calculation functions in **cropgrowdays**. These are:

- `cumulative` calculates cumulative weather data between between two dates, and
- `daily_mean` calculates daily mean of a weather variable between two dates, and
- `growing_degree_days` calculates the growing degree days between two dates as the sum of the difference between daily average temp and a baseline value where the daily average temp is capped, and
- `stress_days_over` calculates the number of days that the maximum temperature is over a baseline value between between two dates.

In addition, several functions are available to calculate the day of year or convert this to a date, namely:

- `day_of_year` calculates day of year from a date, and
- `date_from_day_year` calculates a date from the day of the year and the year, and
- `day_of_harvest` returns day of harvest in the year of sowing which, of course, may be a different year to the year of harvest.

Two functions are available to retrieve SILO weather data from Queensland Government DES longpaddock website.

- `get_silodata` retrieves weather data for one location from the `longpaddock` website, and
- `get_multi_silodata` retrieves weather data for several locations from the `longpaddock` website.

SILO (Scientific Information for Land Owners) is a database of Australian climate data hosted by the Science and Technology Division of the Queensland Government's Department of Environment and Science (DES).

SILO datasets are constructed from Australian Bureau of Meteorology observations and may change due to data correction or improved interpolation or imputation.

## Agrometerological calculations

### Growing Degree Days

The `growing_degree_days` function calculates the sum of degree days for each day $i = 1 \ldots n$. The growing degree days $GDD$ summed over $n$ days are

$$GDD = \sum_{i}^{n} (Tmax_i + Tmin_i)/2 - T_{base}$$

during specified dates for a tibble/data frame of daily weather data. For each day $i$, the maximum temperature is $Tmax_i$ and minimum is $Tmin_i$. Note that the maximum temperature $Tmax$ is capped at `maxt_cap` degrees when calculating average temperature. The defaults are $T_{base} = 5°C$ and $Tmax$ is capped at $Tmax_{cap} = 30°C$. (See [3] or https://farmwest.com/climate/calculator-information/gdd/ [4])

The *gdd* functions in the *pollen* package [5] and in *agroclim* [6] also calculate growing degree days. While these functions do not allow for a fixed number of days, and in the case of *agroclim::gdd* assume a more limited growing season since the function appears to be tailored to grapes, further variations on the formula above as outlined in [7] are available.

### Stress days

`stress_days_over` calculates the number of days when the maximum temperature exceeded a base line `stress_temp` during specified dates for a tibble/data frame of daily weather data. The default `stress_temp` is set at $30°C$.

### Cumulative calculations

`cumulative` calculates the sum total of daily values between two dates from a tibble/data frame of daily weather data. Typically this is used for solar radiation or rainfall.

### Daily means

`daily_mean` calculates the daily average of a variable between two dates from a tibble/data frame of daily weather data. Typically this would be for temperature, rainfall or solar radiation.

## Extracting weather data from a tibble or dataframe

To extract column(s) from a tibble/data frame of daily weather data between two specified dates we use `weather_extract`. Either specify the start and end dates or specify one of these dates and also the number of days after or before, respectively.

## Adding agrometeorological variables to dataframes

We can add agrometeorological variables to a tibble containing crop data using the `tidyverse` functions `map_dbl`, `map_dbl2` and `pmap` to calculate new columns employing the weather data object extracted from the QLD DES website or elsewhere. Use `map_dbl` for one varying date and `map_dbl2` for varying start and end dates. For more than two varying parameters, which may be necessary if for instance our weather object contained multiple locations or sites, then we can use `pmap`. These functions are from the `purrr` library. Alternatively, we could use functions from the `apply` family such as `mapply` from the `base R` package.

These calculations can be time consuming for large datasets. One approach that may prove useful is to use the `furrr` package which is a bridge between `purrr`'s family of mapping functions and `future`'s parallel

processing capabilities. If speed is an issue, then it is worth trying because it is simple to implement. While some tweaking may prove useful, it seems that the defaults work pretty well (see `?future::plan`). After setting the number of workers, then simply replace mapping functions by putting `future_` at the front of the name of the mapping function. For instance, `map2_dbl` is replaced with `future_map2_dbl`.

Further details are provided in the package help and vignettes.

## References


1. Wickham H, Averick M, Bryan J, Chang W, McGowan LD, François R, et al. Welcome to the tidyverse. Journal of Open Source Software. 2019;4: 1686. doi:10.21105/joss.01686
2. Grolemund G, Wickham H. Dates and times made easy with lubridate. Journal of Statistical Software. 2011;40: 1–25. Available: https://www.jstatsoft.org/v40/i03/
3. McMaster GS, Wilhelm WW. Growing degree-days: One equation, two interpretations. Agricultural and Forest Meteorology. 1997;87: 291–300. doi:10.1016/S0168-1923(97)00027-0
4. Anon. GDD [Internet]. Farmwest. 2021. Available: https://farmwest.com/climate/calculator-information/gdd/
5. Nowosad J. Pollen: Analysis of aerobiological data [Internet]. 2019. Available: https://CRAN.R-project.org/package=pollen
6. Serrano-Notivoli R. Agroclim: Climatic indices for agriculture [Internet]. 2020. Available: https://CRAN.R-project.org/package=agroclim
7. Baskerville GL, Emin P. Rapid Estimation of Heat Accumulation from Maximum and Minimum Temperatures. Ecology. 1969;50: 514–517. doi:10.2307/1933912
