# Supplementary material for: Enhancing green bean crop maturity and yield prediction by harnessing the power of statistical analysis, crop records and weather data
Source: PLoS One. 2025 Mar 10;20(3):e0306266. doi: 10.1371/journal.pone.0306266 (PMC11893118; doi:10.1371/journal.pone.0306266)
Supplement: S2 File — (PDF) [file pone.0306266.s002.pdf]

S2 File 2. Example models for prediction of growth periods and fresh bean yield

The following are a few linear statistical models, which have some use for prediction. The exact model is not as important as the insight of agro-met variables affecting growth. Variable names are described in Support Table S1

Modelling of day of petal fall and growing periods using observed agronomic and derived agro-meteorological variables

Selected linear Models	PREDICT RESPONSE	Adj R Sq	RMSE	Unit of response
dofy_pf ~ dofy_sow, subset "Autumn"	day of Petal Fall (Autumn)	**0.9758**	3.0930	Day (fin year) of petal fall
dofy_pf ~ dofy_sow, subset "Spring"	day of Petal Fall (Spring)	**0.9756**	3.4550	Day (fin year) of petal fall
gsfy_veg ~ dofy_sow + gddays5_sow_pf + daily_rad_sow_pf	Veg (both seasons)	**0.6978**	2.4164	Duration vegetative period
gsfy_veg ~ dofy_sow + gddays5_sow_pf + avmint_sow_pf + daily_rad_sow_pf + cum_rad_sow_pf + avmaxt_sow_28d + avmaxt_sow_7d + daily_rad_sow_28d + avmint_sow_28d + awp_sow_28d + avmint_pf_ah + awp_pf_ah + avmaxt_sow_14d + avmint_sow_14d, subset "Autumn"	Veg (Autumn season)	**0.9921**	0.2769	Duration vegetative period (in days)
gsfy_veg ~ dofy_sow + gddays5_sow_pf + gddays5_sow_28d + avmaxt_sow_pf + avmint_sow_28d + avmaxt_sow_28d + cum_rad_sow_pf + daily_rad_sow_pf + gddays5_sow_14d + awp_sow_28d + avmint_pf_ah + avmaxt_sow_7d + daily_rad_sow_28d + daily_rad_sow_21d + avmaxt_sow_21d + awp_sow_21d, subset "Spring"	Veg (Spring season)	**0.9925**	0.3339	Duration vegetative period (in days)
gsfy_pod ~ dofy_sow + gddays5_pf_ah + stressdays_30_pf_5d + stressdays_30_pf_ah + avmint_sow_ah + stressdays_27_5_pf_15d + stressdays_30_pf_15d	Pod fill (both seasons)	**0.9215**	0.915	Duration of pod fill (in days)
gsfy_pod ~ dofy_sow + gddays5_pf_15d + gddays5_pf_ah + stressdays_30_pf_15d + avmaxt_pf_ah + avmint_pf_ah + avmint_pf_15d + avmaxt_pf_15d + stressdays_27_5_pf_15d + stressdays_27_5_pf_ah + cum_rad_pf_5d + stressdays_30_pf_5d + daily_rad_pf_ah + cum_rad_pf_15d	Pod fill (both seasons)	**0.9869**	0.374	Duration of pod fill (in days)
gsfy_tot ~ dofy_sow + cum_rad_sow_pf + daily_rad_sow_pf + stressdays_27_5_sow_pf, subset "Autumn"	Total (Autumn)	**0.6874**	1.91	Duration of total growing season (in days)
gsfy_tot ~ cum_rad_sow_pf + daily_rad_sow_pf + avmaxt_sow_pf + gddays5_sow_pf + avmint_sow_pf, subset "Spring"	Total (Spring)	**0.7641**	1.071	Duration of total growing season (in days)

Predicting day of petal fall in SE Queensland from sowing date for Autumn and Spring seasons

Predicting petal fall in SE Queensland is strongly related to the date of sowing Fig 1. A quadratic component was found to assist in improving the fit.

Predicting total growing period to green bean harvest

This model that it involves the fresh weight of immature bean pod, but one that is at optimum fresh weight and quality as a fresh bean harvest. The statistical model at this point takes into account the day of sowing and the seasons in SE Queensland. The fresh weight is somewhat imnprecisely measured, and quality is not measured.

```
- gsfy_tot ~  fy_season + dofy_sow + I(dofy_sow^2) + dofy_pf
```

Models using derived agro-meteorological variables

Key variables in the vegetative prediction: day of sowing, thermal time and daily radiation. Key variables in the pod fill period prediction: season of sowing, growing degree days, stress days and radiation. For the total period prediction - season of sowing and radiation variables.

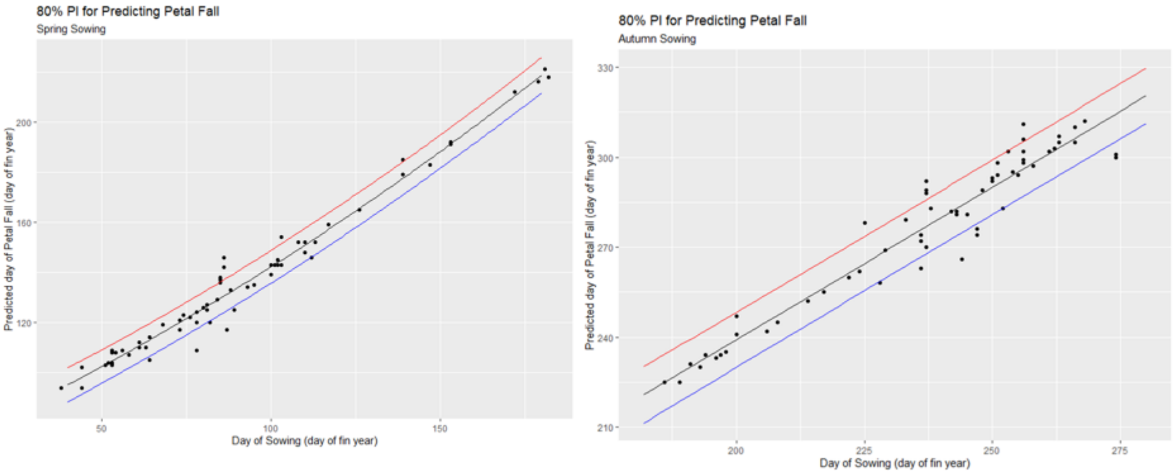

Figure 1:

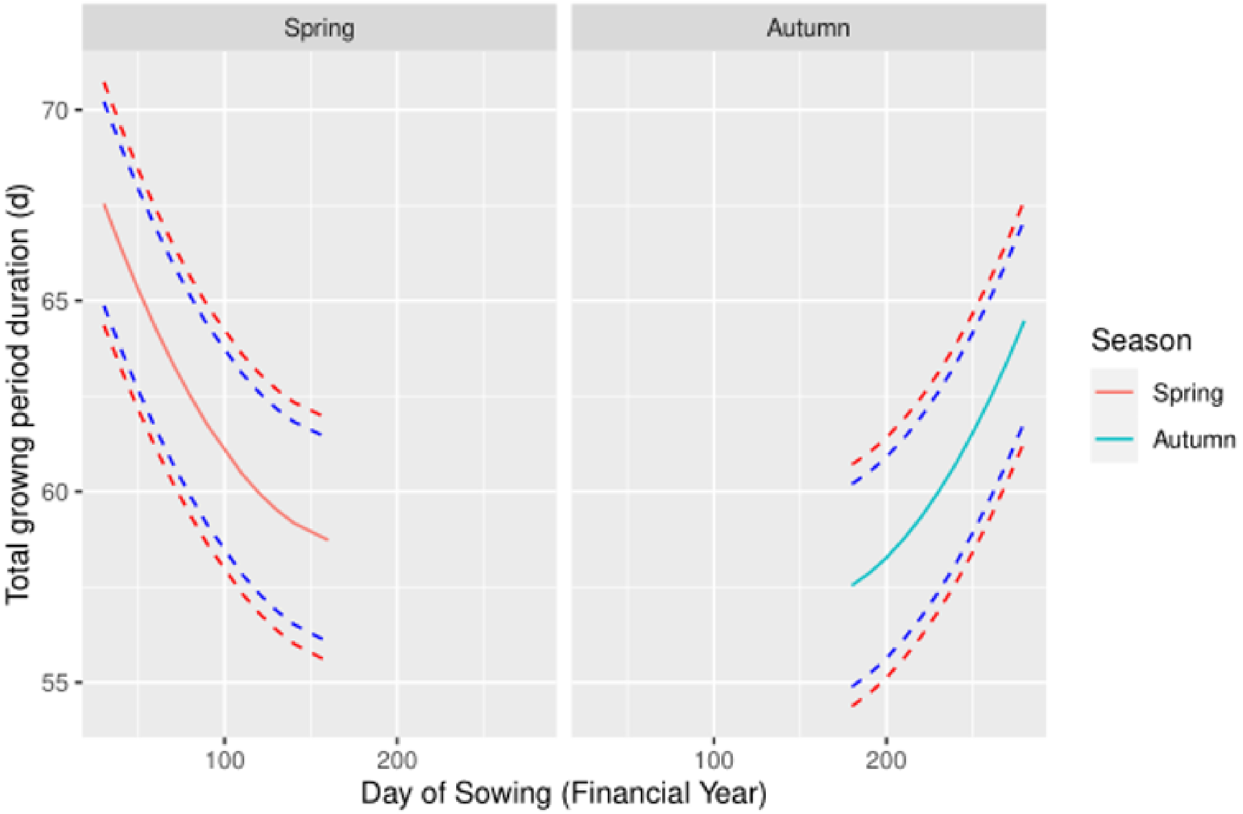

Figure 2:

```
Vegetative duration          Adjusted RSq        0.6727
gsfy_veg ~ dofy_sow + gddays5_sow_pf + daily_rad_sow_pf

Pod filling duration          Adjusted RSq          0.7079
gsfy_pod ~ fy_season * (daily_rad_pf_ah + gddays5_pf_ah + stressdays_30_pf_ah)

Total sowing to actual harvest duration       Adjusted RSq  0.8900
gsfy_tot ~ fy_season * (daily_rad_sow_28d + daily_rad_pf_ah + cum_rad_sow_pf)
```

Models using for the sampled plots

```
VEGETATIVE DURATION
$modv1$,    Adjusted R-squared:  0.9925
MODEL: gsfy_veg ~ dofy_sow + gddays5_sow_pf + avmint_sow_pf +
       daily_rad_sow_pf + cum_rad_sow_pf + avmaxt_sow_28d + avmaxt_sow_7d +
    daily_rad_sow_28d + avmint_sow_28d + avvp_sow_28d + avmint_pf_ah +
       avvp_pf_ah + avmaxt_sow_14d + avmint_sow_14d

$modv2$,     Adjusted R-squared:  0.6978
MODEL:  gsfy_veg ~ dofy_sow + gddays5_sow_pf + daily_rad_sow_pf

POD FILLING DURATION
$modp1$,    Adjusted R-squared:  0.5937
MODEL: gsfy_pod ~ dofy_sow + avmaxt_pf_ah + gddays5_pf_ah +
    gddays5_pf_15d + stressdays_30_pf_ah + avmint_pf_ah + avmint_pf_15d +
       avmaxt_pf_15d + stressdays_30_pf_5d + avvp_pf_15d + stressdays_27_5_pf_15d +
    stressdays_27_5_pf_ah + cum_rad_pf_5d + avmaxt_pf_10d + avmint_pf_5d +   daily_rad_pf_ah

$modp2$,  Adjusted R-squared:  0.9869
MODEL: gsfy_pod ~ dofy_sow + gddays5_pf_15d + gddays5_pf_ah +
     stressdays_30_pf_15d + avmaxt_pf_ah + avmint_pf_ah + avmint_pf_15d +
     avmaxt_pf_15d + stressdays_27_5_pf_15d + stressdays_27_5_pf_ah +
     cum_rad_pf_5d + stressdays_30_pf_5d + daily_rad_pf_ah + cum_rad_pf_15d

 $modp3$ ,    Adjusted R-squared:  0.9215
MODEL: gsfy_pod ~ dofy_sow + gddays5_pf_ah + stressdays_30_pf_5d +
    stressdays_30_pf_ah + avmint_sow_ah + stressdays_27_5_pf_15d

  $modp4$,   Adjusted R-squared:  0.7817
MODEL: gsfy_pod ~ dofy_sow + gddays5_pf_ah + stressdays_30_pf_5d +  stressdays_27_5_pf_15d

$modp5$   Adjusted R-squared:  0.5937
    MODEL: gsfy_pod ~ dofy_sow + stressdays_30_pf_5d + stressdays_27_5_pf_15d

TOTAL DURATION
$modtot1$   Adjusted R-squared:  0.6976
MODEL:   gsfy_tot ~ dofy_sow + cum_rad_sow_pf + daily_rad_sow_pf +
        stressdays_27_5_sow_pf + avvp_sow_pf + gddays5_sow_pf + avmaxt_sow_pf
```
