# Supplementary material for: Enhancing green bean crop maturity and yield prediction by harnessing the power of statistical analysis, crop records and weather data
Source: PLoS One. 2025 Mar 10;20(3):e0306266. doi: 10.1371/journal.pone.0306266 (PMC11893118; doi:10.1371/journal.pone.0306266)
Supplement: S1 Table — (PDF) [file pone.0306266.s003.pdf]

Table S1. Agro-meteorological variables made specifically for the actual sowing dates

The following are variables in the statistical models, with the naming system employed to make the derived variables. The code from the R package is shown above the variable. At the end are the phenological variables and the dates and duration of growing period variables. See *cropgrowdays* R package for full compliment of code and the extract of daily meteorological data for past years from SILO [1].

1 Agro-meteorological derived variables

1.1 The prefixes indicate the variable

*gddays_*	growing degree days (base 5 and cap 30 C for our data)
*avmint_*	average daily minimum temperature
*avmaxt_*	average daily maximum temperature
*cum_rad_*	cumulative radiation over a period
*daily_rad_*	average daily radiation over a period
*avvp_*	average daily vapour pressure
*stressdays_*27_5_	number of days with temperatures over 27.5 C in a period
*stressdays_*30_	number of days with temperatures over 30 C in a period

1.2 The suffixes indicate the period for the calculation

_sow_pf	sowing to petal fall (vegetative period)
_sow_ah	sowing to actual harvest (total growing period)
_pf_ah	petal fall to actual harvest (pod fill period)
*_sow_*7*d*	sowing to 7 days post sowing
*_sow_*14*d*	sowing to 14 days post sowing
*_sow_*21*d*	sowing to 21 days post sowing
*_sow_*28*d*	sowing to 28 days post sowing
*_pf_*5	petal fall to 5 days post petal fall
*_pf_*10*d*	petal fall to 10 days post petal fall
*_pf_*15*d*	petal fall to 15 days post petal fall

[1] longpaddock Queensland Government DES, https://www.longpaddock.qld.gov.au)

2 KEY variables

Use data file sites_105_years extracted weather to match exact year and date of sowing.

2.1 Thermal Time

gddays5_sow_pf
gddays5_pf_ah
gddays5_sow_ah
gddays5_sow_7d
gddays5_sow_14d
gddays5_sow_21d
gddays5_sow_28d
gddays5_pf_5
gddays5_pf_10
gddays5_pf_15

2.2 Min Temp for periods

avmint_sow_pf
avmint_sow_ah
avmint_pf_ah
avmint_post_sow_7d
avmint_post_sow_14d
avmint_post_sow_21d
avmint_post_sow_28d
avmint_post_pf_5
avmint_post_pf_10
avmint_post_pf_15

2.3 Max Temp for periods

avmaxt_sow_pf
avmaxt_sow_ah
avmaxt_pf_ah
avmaxt_post_sow_7d
avmaxt_post_sow_14d
avmaxt_post_sow_21d
avmaxt_post_sow_28d
avmaxt_post_pf_5
avmaxt_post_pf_10
avmaxt_post_pf_15

2.4 Cumulative Radiation for periods

cum_rad_sow_pf
cum_rad_pf_ah
cum_rad_sow_ah
cum_rad_post_sow_7d
cum_rad_post_sow_14d
cum_rad_post_sow_21d
cum_rad_post_sow_28d
cum_rad_post_pf_5
cum_rad_post_pf_10
cum_rad_post_pf_15

2.5 Daily Radiation for periods

daily_rad_sow_pf
daily_rad_pf_ah
daily_rad_sow_ah
daily_rad_post_sow_7d
daily_rad_post_sow_14d
daily_rad_post_sow_21d
daily_rad_post_sow_28d
daily_rad_post_pf_5
daily_rad_post_pf_10
daily_rad_post_pf_15

2.6 Stress days over a 27.5 C temperature

stressdays_27_5_pf_ah
stressdays_27_5_sow_pf
stressdays_27_5_sow_ah
stressdays_27_5_podf_5
stressdays_27_5_podf_10
stressdays_27_5_podf_15

2.7 Stress days over a 30 C temperature

stressdays_30_pf_ah
stressdays_30_sow_pf
stressdays_30_sow_ah
stressdays_30_podf_5
stressdays_30_podf_10
stressdays_30_podf_15

2.8 Phenological variables

gsfy_veg	length of vegetative period in days
gsfy_pod	length of pod filling period in days
gsfy_tot	length of total growing season in days
fy_season	season (Autumn, Spring, Middle)

2.9 Dates and durations

These transformations are also available in our *cropgrowdays* package.

```
day_of_year(x,
type = c("calendar", "financial", "other"),
return_year = FALSE,
base = NULL)
```

```
date_from_day_year(day,
year,
type = c("calendar", "financial", "other"),
base = NULL)
```

date_sow	date format (eg. 2017-02-2)
date_pf	date format
date_ah	date format
doy_sow	day of year format (1 Jan = 1)
doy_pf	day of year format
doy_ah	day of year format
dofy_sow	day of financial year format (1 July = 1)
dofy_pf	day of financial year format
dfoy_ah	day of financial year format
